# Long term effect of a medical emergency team on cardiac arrests in a teaching hospital

**DOI:** 10.1186/cc3906

**Published:** 2005-11-16

**Authors:** Daryl Jones, Rinaldo Bellomo, Samantha Bates, Stephen Warrillow, Donna Goldsmith, Graeme Hart, Helen Opdam, Geoffrey Gutteridge

**Affiliations:** 1 Clinical Fellow, Department of Intensive Care, Alfred Hospital, Commercial Road, Prahran, Melbourne, Victoria 3181, Australia; 2 Director of Research, Department of Intensive Care and Department of Surgery (Melbourne University), Austin Hospital, Studley Road, Heidelberg, Melbourne, Victoria 3084, Australia; 3 Research Nurse, Department of Intensive Care and Department of Surgery (Melbourne University), Austin Hospital, Studley Road, Heidelberg, Melbourne, Victoria 3084, Australia; 4 Staff Specialist, Department of Intensive Care and Department of Surgery (Melbourne University), Austin Hospital, Studley Road, Heidelberg, Melbourne, Victoria 3084, Australia; 5 Research Nurse, Department of Intensive Care and Department of Surgery (Melbourne University), Austin Hospital, Studley Road, Heidelberg, Melbourne, Victoria 3084, Australia; 6 Staff Specialist, Department of Intensive Care and Department of Surgery (Melbourne University), Austin Hospital, Studley Road, Heidelberg, Melbourne, Victoria 3084, Australia; 7 Staff Specialist, Department of Intensive Care and Department of Surgery (Melbourne University), Austin Hospital, Studley Road, Heidelberg, Melbourne, Victoria 3084, Australia; 8 Staff Specialist, Department of Intensive Care and Department of Surgery (Melbourne University), Austin Hospital, Studley Road, Heidelberg, Melbourne, Victoria 3084, Australia

## Abstract

**Introduction:**

It is unknown whether the reported short-term reduction in cardiac arrests associated with the introduction of the medical emergency team (MET) system can be sustained.

**Method:**

We conducted a prospective, controlled before-and-after examination of the effect of a MET system on the long-term incidence of cardiac arrests. We included consecutive patients admitted during three study periods: before the introduction of the MET; during the education phase preceding the implementation of the MET; and a period of four years from the implementation of the MET system. Cardiac arrests were identified from a log book of cardiac arrest calls and cross-referenced with case report forms and the intensive care unit admissions database. We measured the number of hospital admissions and MET reviews during each period, performed multivariate logistic regression analysis to identify predictors of mortality following cardiac arrest and studied the correlation between the rate of MET calls with the rate of cardiac arrests.

**Results:**

Before the introduction of the MET system there were 66 cardiac arrests and 16,246 admissions (4.06 cardiac arrests per 1,000 admissions). During the education period, the incidence of cardiac arrests decreased to 2.45 per 1,000 admissions (odds ratio (OR) for cardiac arrest 0.60; 95% confidence interval (CI) 0.43–0.86; *p *= 0.004). After the implementation of the MET system, the incidence of cardiac arrests further decreased to 1.90 per 1,000 admissions (OR for cardiac arrest 0.47; 95% CI 0.35–0.62; *p *< 0.0001). There was an inverse correlation between the number of MET calls in each calendar year and the number of cardiac arrests for the same year (r^2 ^= 0.84; *p *= 0.01), with 17 MET calls being associated with one less cardiac arrest. Male gender (OR 2.88; 95% CI 1.34–6.19) and an initial rhythm of either asystole (OR 7.58; 95% CI 3.15–18.25; *p *< 0.0001) or pulseless electrical activity (OR 4.09; 95% CI 1.59–10.51; *p *= 0.003) predicted an increased risk of death.

**Conclusion:**

Introduction of a MET system into a teaching hospital was associated with a sustained and progressive reduction in cardiac arrests over a four year period. Our findings show sustainability and suggest that, for every 17 MET calls, one cardiac arrest might be prevented.

## Introduction

Despite advances in medical technology and the introduction of cardiac arrest teams, the mortality of in-hospital cardiac arrests remains high (approximately 85% [1]). Up to 80% of patients suffering cardiac arrests have signs of physiological instability (alterations in the commonly measured vital signs) in the 24 hour period before the event [2-4]. In response to this observation, hospitals are increasingly implementing specialized teams (variably named medical emergency teams (METs), rapid response teams, or outreach teams) to identify, review and treat unstable ward patients in the early phase of deterioration, with the goal of preventing cardiac arrests [5-8].

METs have been shown to reduce the incidence of cardiac arrests in hospitalized patients in short-term before-and-after studies [5-7]. DeVita and co-workers [8] have recently reported a 17% reduction in cardiac arrests in the 1.8 years after increased use of the MET service in a teaching hospital [8]. The effectiveness of METs in achieving or sustaining this outcome for periods greater than this has not been shown to date.

Since the introduction of the MET service in our hospital, we have conducted an educational campaign to improve awareness in an attempt to increase use of the service. Sustained system change, however, requires a strong organizational commitment to safety [9] as well as continued education and awareness-raising activities. Institutionalization of system change may fail because of turnover of key employees [10] such as doctors and nurses. This may result in the introduction of new staff who are unfamiliar with the MET concept [8,11]. Thus, there is uncertainty that the MET system will continue to deliver any benefits that might have been demonstrated immediately after its introduction. In this study, we analyzed the incidence of cardiac arrests in the four years following the introduction of the MET service.

## Materials and methods

### The hospital

The Austin Hospital is a 400 bed acute care hospital affiliated with the University of Melbourne. The hospital provides services such as cardiothoracic surgery and neurosurgery and is also the referral center for acute spinal injuries and liver transplantation for the state of Victoria. The intensive care unit (ICU) has 21 beds and receives approximately 2,000 admissions per year.

### Ethics approval

Approval was obtained from the Institution's Ethics Committee for implementation of the MET system and collection of the data related to it. The need for informed consent was waived by the committee.

### Medical emergency responses

The acute care hospital has two levels of emergency response. The traditional 'Code Blue' call is intended for resuscitation of cardiac arrests and other sudden life-threatening medical emergencies. It consists of an anesthetic fellow, a coronary care fellow and nurse, an ICU fellow and nurse, as well as the Medical fellow of the receiving unit of the day. The MET is intended to review all medical emergencies other than cardiac arrests, and has been described in detail previously [7,12]. It consists of an ICU fellow and nurse, as well as the Medical fellow of the receiving unit of the day. It can be activated by any member of hospital staff according to pre-determined criteria that are based primarily on abnormalities of vital signs and clinical status. Specifically, the criteria include acute changes in heart rate (<40 or >130 beats/minute), systolic blood pressure (<90 mmHg), respiratory rate (<8 or >30 breaths/minute), oxygen saturation (<90% despite oxygen therapy) or conscious state. Any staff member can also initiate a MET review if they are worried about the patient for any other reason.

### Study design

The study is a prospective before-and-after interventional trial. All patients admitted to the hospital for at least one night were considered as participants. The period 1 January 1999 to 31 August 1999 was the control or 'before' period. In the educational phase preceding implementation of the MET (1 September 1999 to 31 August 2000), detailed education and information sessions where held with all members of hospital staff. The period 'after' the implementation of the MET included the interval spanning from 1 September 2000 to 31 October 2004.

### Data on admissions, cardiac arrests and MET calls

A log book of all emergency calls is maintained by the switchboard operators and contains details of the date and time of the call as well as the ward from which the call originated. Standardized case report forms on Code Blue calls are maintained independently by both the coronary care unit and ICU. Details from the switchboard log regarding time, date and ward calling the Code Blue calls were cross referenced with the case report forms and the ICU admission database. Calls were designated as 'documented cardiac arrest', 'not cardiac arrest' or 'insufficient data'. Information was confirmed by two investigators by analysis of the case report form and/or analysis of the patients file. A cardiac arrest was defined as the sudden onset of all of the following: no palpable pulse; no measurable blood pressure; no responsiveness; and the commencement of basic life support. Calls from the coronary care unit, operating room and emergency room (acute care areas) were not considered for analysis, as were calls in which the patient had a documented 'not for resuscitation' order prior to the call. To minimize bias in favor of the MET system, calls designated 'insufficient data' were still considered as being 'true cardiac arrests' in the educational phase and in the period after the introduction of the MET. Calls designated as 'not cardiac arrest' were then classified into one of nine subgroups (faint, seizure, haematemesis and melena, surgical bleeding, respiratory distress, altered conscious state, hypotension, arrhythmia, chest pain).

### Outcome measures

The number of cardiac arrests in each of the study periods ('before MET', 'educational phase preceding MET' and 'after MET implementation') was quantified and related to the number of hospital admissions lasting at least 24 hours for the same period. Information on the age, gender and initial documented rhythm were obtained from the case report forms. Data on patient hospital outcome and destination of hospital discharge were obtained from the hospital electronic database.

### Statistical analysis

Data on the incidence of cardiac arrests during the three periods of the study were compared using the χ^2 ^test and analyzed using MS Windows Stat-view (Abacus Concepts, Berkeley, CA, USA). Correlation between levels of MET service activation and the incidence of cardiac arrests was analyzed using the Spearman-rank test.

We performed multivariate logistic regression analysis using death as the dependent variable and patient gender, age, parent unit, primary rhythm and time of cardiac arrest as independent variables. Data were analyzed using SPSS (version 12, SPSS Inc, Chicago, IL, USA) and results are presented as odds ratio (OR) with 95% confidence intervals (CI). A *p* value of <0.05 was considered statistically significant.

The number of MET reviews associated with a decrease of one cardiac arrest was estimated by calculating the reciprocal of the line of regression of the association between the level of MET service activation and the incidence of cardiac arrests.

## Results

### Characteristics of Code Blue calls

Compared to the period before the MET, there was an initial reduction in the number of Code Blue calls during the education phase (OR for Code Blue call 0.75; 95% CI 0.57–0.98; *p *= 0.033). After the implementation of the MET, however, there was a further significant reduction in Code Blue calls (OR for Code Blue call 0.51; 95% CI 0.41–0.64; *p *< 0.0001) (Table 1). At least 30% of Code Blue calls were made for indications other than cardiac arrest. Specifically, the major reasons for such Code Blue calls were respiratory distress, hypotension, altered Glasgow coma score, seizure and a simple collapse or faint (Table 1).

In addition, there was no significant difference in the proportion of patients in whom a Code Blue call was made despite a prior documented 'not for resuscitation' order (OR 1.40; 95% CI 0.46–4.23; *p *= 0.57).

### Impact of MET implementation on the incidence of cardiac arrests

When the Code Blue calls classified as 'insufficient data' were included as true cardiac arrests for the education and post-MET implementation period, the number of cardiac arrests before the MET, during the educational phase preceding the MET, and after the implementation of the MET was 66, 62 and 198, respectively (Table 1). The overall process of introduction of the MET was associated with a statistically significant reduction in cardiac arrests when compared with the period before the MET (multiple χ^2 ^*p *< 0.0001). During the educational phase preceding the MET, the OR for risk of cardiac arrest was 0.60 (95% CI 0.43–0.86; *p *= 0.004) when compared with the period before the MET. After the introduction of the MET system, the OR of cardiac arrest was 0.47 (95% CI 0.35–0.62; *p *< 0.0001) when compared to the period before MET implementation and 0.77 (95% CI 0.58–1.03; *p *= 0.078) when compared to the educational phase. In the year 2004, there was a further reduction to a rate of 1.3 arrests per 1,000 admissions, such that the OR for cardiac arrest was 0.31 (95% CI 0.20–0.48; *p *< 0.0001) when compared with the period before the MET and 0.51 (95% CI 0.32–0.80; *p *= 0.003) compared with the educational phase preceding the MET.

### Characteristics and outcome of documented cardiac arrests

There were 279 documented cardiac arrests during the study period (Table 2). The majority of these patients were male, and their average age was 71.3 years. Patients from five of the 20 units (general medicine, respiratory medicine, cardiology, renal medicine and haematology/oncology) comprised 62% of all calls. The majority (170/228, 74.6%) of patients who died following a cardiac arrest did so at the time of the cardiac arrest. The most common rhythms present on arrival of the cardiac arrest team were asystole (122/279, 43.7%) and pulseless electrical activity (70/279, 25.1%). The survival rate following a cardiac arrest was not affected by the introduction of the MET (OR for survival 0.60; 95% CI 0.30–1.21; *p *= 0.15) (Table 2).

Several independent variables were shown to have an impact on outcome following cardiac arrest (Table 3). Male gender and an initial rhythm of asystole or pulseless electrical activity were associated with an increased risk of death following cardiac arrests. In addition, none of the 23 patients admitted under haematology/oncology survived a documented cardiac arrest in the study period.

Predictors of a decreased risk of death following cardiac arrest included admission under cardiothoracic surgery, spinal cord injuries unit, cardiology unit, or an arrest between the hours of 08:00 and 10:00.

**Figure 1:**
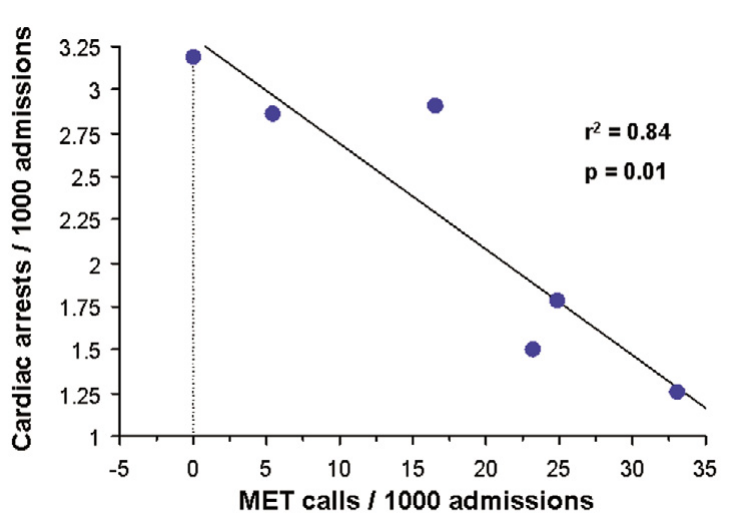
Correlation between the number of medical emergency team (MET) calls and the rate of cardiac arrests between 1999 and 2004.

There was an inverse correlation (r^2 ^= 0.84, *p *= 0.01) between the number of MET calls per 1,000 admissions in each calendar year and the number of cardiac arrests per 1,000 admissions over the corresponding period (Figure 1). The gradient of the line of regression for this correlation was -0.061, suggesting that for every 17 MET calls (1/0.061) there was an associated decrease of one cardiac arrest.

## Discussion

We conducted a study to test whether the ongoing use of a MET system could be associated with a sustained reduction in the incidence of cardiac arrests over the four years after its introduction. We found that education alone decreased the incidence of cardiac arrests. However, the risk of cardiac arrest further decreased after the introduction of the MET and remained lower over time.

We also found an inverse association between levels of MET use and the risk of occurrence of cardiac arrests, suggesting a 'dose effect' of the MET on cardiac arrests.

Implementation of MET systems has previously been shown to reduce the incidence of cardiac arrests in hospital patients in a number of short term before-and-after studies [5-7]. DeVita and co-workers [8], however, reported that increasing the use of an existing MET was associated with a 17% reduction in the incidence of cardiac arrests in the subsequent 1.8 years to a rate of 5.4 per 1,000 admissions. In the present study, the incidence of cardiac arrests was 4.06 per 1,000 admissions before the introduction of the MET. After the introduction of the MET, the incidence of cardiac arrests was 1.90 per 1,000 admissions (53% reduction) and this decreased to 1.3 per 1,000 admissions (69% reduction) in 2004. These later results suggest a greater reduction in the incidence of cardiac arrests than previously reported following the introduction of a MET service.

Sustained system change requires strong organizational commitment to safety [9]. System change may fail to be institutionalized because of a turnover of key employees [10], resulting in new doctors and nurses unfamiliar with the MET system concept [8,11]. Prior to the implementation of the MET service at our hospital, a 12 month period of preparation and education was undertaken (educational phase). Since the introduction of the MET service we have provided ongoing education to all existing and new staff members. This approach appears to have been successful in producing a sustained and progressive reduction in the incidence of cardiac arrests at our hospital.

In addition to the reduction in incidence of cardiac arrests demonstrated after the introduction of the MET service, we have found an inverse association between levels of MET activation and the incidence of cardiac arrest. This suggests an association between increasing use of the MET and a reduction in cardiac arrests. From this association, we estimate that for every 17 MET calls a single cardiac arrest may be prevented.

Multivariate logistic regression analysis revealed male gender and a primary rhythm of asystole or pulseless electrical activity as predictors of increased risk of death following cardiac arrest. Predictors of improved outcome following cardiac arrest included admission under cardiothoracic surgery, the spinal cord injury unit, or cardiology. This improved outcome may be secondary to increased levels of monitoring leading to earlier detection of cardiac arrests in these patients, or may suggest greater reversibility of the underlying process leading to the arrest (for example, myocardial ischemia or altered autonomic tone with spinal cord injury). Improved outcome for cardiac arrests occurring between 08:00 and 10:00 may relate to increased levels of staffing and earlier detection of events.

Our study has several strengths and limitations. It is a prospective before-and-after study that demonstrates a progressive and dose-dependent reduction in the annual incidence of cardiac arrests in a large teaching hospital in a study period including over 145,000 admissions. It is not randomized, blinded or placebo-controlled, however, and only represents the findings of a single center. A large cluster-randomized multi-centre study of the effect of the MET on cardiac arrests in 23 Australian hospitals (the MERIT study) has been recently published [13]. The incidence of cardiac arrests in the 12 hospitals randomized to receive MET implementation was not statistically different to that in the 11 hospitals randomized to continue the existing form of cardiac arrest teams. This study involved an education and preparation period of only four months, however, and assessed the impact of the introduction of MET reviews over six months only. In addition, the average call rate (cardiac arrests and MET calls) was reported as only 8.3 calls per 1,000 admissions. This call rate is one-fifth of our current call rate. These observations suggest that time may be required before a MET system can show its full effectiveness in reducing the incidence of cardiac arrests. The benefits from other system changes, such as trauma services [14], have also been shown to take some time to mature. These results also suggest that the 'dose' of MET reviews (calls per 1,000 patients) may have an impact on patient outcome.

The second limitation of our study relates to the inclusion of episodes of insufficient data. In analyzing the effectiveness of the education process and MET service, however, we have assumed that these events represented a true cardiac arrest. In doing this, we would have actually underestimated the effectiveness of the MET system, as such calls likely inflated the true cardiac arrest call value after implementation of the MET.

Our study does not reveal the mechanisms responsible for the reduction of the cardiac arrests. It is possible that the observed reduction in the incidence of cardiac arrest may be due to the education of staff alone, as the incidence of cardiac arrests in the educational phase decreased by 40% compared with the period before the introduction of the MET. However, the incidence of cardiac arrests after the introduction of the MET was 23% lower than during the education and preparation period, and has continued to fall with increasing use of the MET. This suggests education and awareness together with a system to promptly review unwell ward patients work synergistically in reducing the incidence of cardiac arrests.

It is possible that our favorable findings were due to a high incidence of cardiac arrests in the control period or an abnormally low seasonal incidence in the intervention period. Australian data [16] show an incidence ranging from 3.6 to 5.1 per 1,000 admissions. Recent data from the MERIT study, which included several smaller hospitals with patients of limited acuity and counted all day visits (no overnight stay) as admissions, showed a pre-intervention cardiac arrest rate of 2.08 cases per 1,000 admissions [13]. Our incidence of cardiac arrests was 3.2 per 1,000 overnight hospital admissions during the control period.

We studied the MET within a single institution. Its findings might not apply to other hospitals. Institution specific heuristics and unique administrative features may have lent themselves to making the impact of the MET approach greater in our institution than in others. However, our institution has all the organizational, structural and logistic features of a typical tertiary referral hospital. The way our MET was configured might differ from the way other institutions implement such a service [13,15]. Whether organizing the MET service in different ways has an impact on its efficacy remains unknown. We believe that our approach is simple and low cost. It is also possible that the decrease in cardiac arrests was secondary to some other improvements in patient care during the period that separated the control from the intervention period. There were no changes in the structure, referral pattern or activity of our hospital, however, as supported by the total number of admissions during the two study periods, which remained unchanged. Furthermore, there were no changes in 'not for cardio-pulmonary resuscitation' policy, hospital admission policy, discharge practices or surgical case mix during the study. We are not aware of any improvements or advances in medical or surgical treatment that could explain a greater than 60% reduction in cardiac arrests and a 25% reduction in overall mortality.

## Conclusion

The introduction of an ICU-based MET service, in conjunction with a detailed education program aimed at increasing its use, was associated with a sustained and progressive reduction in the incidence of cardiac arrests in the four years following its implementation. There was an inverse correlation between the level of activation of the MET service and the incidence of cardiac arrests, suggesting either a 'dose effect' or a 'maturation effect'.

## Key messages

• 	Education and preparation for the MET system decreased the incidence of cardiac arrests.

• 	Introduction of the MET system further reduced the incidence of cardiac arrests.

• 	The incidence of cardiac arrests was inversely proportional to the number of MET calls and is continuing to decrease four years after the introduction of the system as MET calls continue to increase.

• 	There appears to be a 'dose effect' such that, for each 17 MET calls, one less cardiac arrest occurs.

• 	If education and staff awareness are maintained after initial introduction, the MET system continues to increase in efficacy. Short-term studies may underestimate its impact.

## Abbreviations

CI = confidence interval; ICU = intensive care unit; MET = medical emergency team; OR = odds ratio.

## Competing interests

The authors declare that they have no competing interests.

## Authors' contributions

DJ planned and designed the study, wrote the article and conducted data collection and analysis. SB, SW and DG assisted with data collection and analysis and with review of the final draft. HO, GH and GG assisted in the design and planning of the study and with review of the final draft. RB supervised study planning, design, execution and analysis and co-wrote and edited the manuscript

## Figures and Tables

**Table 1:** Characteristics of Code Blue calls over the study period

	Pre-MET^a^	Education phase^a^	Post-MET implementation^a^
Code Blue calls	100	116	327
Documented cardiac arrests	66	51	162
Missing data	1	11	36
NFR patients (arrests in NFR patients)	4 (2)	7 (5)	18 (17)
Not cardiac arrests	31	49	112
Faint	4	4	11
Seizure	7	2	11
H&M	1	3	5
Surgical bleeding	2	0	10
Respiratory distress	13	19	38
Change GCS	1	8	13
Hypotension	2	10	17
Arrhythmia	1	2	7
Chest pain	0	1	0
Admissions	16,246	25,216	104,001
Cardiac arrests^b^			
Number	66	62	198
Number/1,000 admissions	4.06	2.45	1.90
Odds ratio for risk of cardiac arrest compared to before MET		0.60 (95% CI 0.43–0.86) *p *= 0.004	0.47 (95% CI 0.35–0.62) *p *< 0.0001

^a^The three study periods cover the dates: pre-MET, 1 January 1999 to 31 August 1999; education phase, 1 September 1999 to 31 August 2000; post-MET implementation, 1 September 2000 to 31 October 2004. ^b^Cardiac arrests represent the combined number of 'documented cardiac arrests' and 'insufficient data' for 'during' and 'after' MET implementation phases. Odds ratios are for comparisons with 'before' MET data. GCS, Glasgow coma score; H&M, hematemesis and melena; NFR, not for resuscitation.

**Table 2:** Characteristics of documented cardiac arrests

	Pre-MET^a^	Education phase^a^	Post-MET^a^
Cardiac arrests	66	51	162
Male	41	44	104
Age (years)	73.4	70.5	70.8
Parent unit			
General medicine	19 (4)	11 (1)	45 (3)
Respiratory	6 (1)	5 (1)	13 (3)
Cardiology	1 (0)	5 (2)	19 (8)
Nephrology	8 (3)	3 (0)	16 (2)
Gastroenterology	3 (0)	1 (0)	2 (0)
Neurology	0	4 (0)	7 (1)
Hematolgy/Oncology	2 (0)	7 (0)	14 (0)
General surgery	5 (0)	7 (2)	9 (1)
Vascular surgery	8 (2)	1 (0)	2 (0)
Neurosurgery	3 (0)	1 (0)	7 (3)
Thoracic surgery	2 (2)	1 (1)	4 (0)
Cardiac surgery	5 (3)	3 (2)	9 (3)
Orthopedic surgery	2 (1)	1 (0)	8 (0)
Spinal surgery	1 (0)	0	2 (2)
Liver transplantation	1 (0)	0	3 (0)
Other^b^	0	1 (0)	2 (0)
Survivors (% total)	16 (24.2)	9 (17.6)	26 (16.0)
Home	10	5	20
Rehab	5	3	6
Nursing home	1	1	0
Died	50	42	136
Died arrest	33	27	110
Died <24 h	10	5	9
Died >24 h	7	10	17
Primary rhythm			
VF	8 (3)	10 (1)	20 (9)
VT	4 (2)	6 (3)	15 (6)
Asystole	30 (1)	18 (2)	74 (6)
PEA	15 (4)	13 (2)	42 (3)
SVT	2 (2)	0	3 (1)
Other	7 (4)	4 (1)	8 (1)

Data are expressed as total number with number surviving in parentheses. ^a^The three study periods cover the dates: pre-MET, 1 January 1999 to 31 August 1999; education phase, 1 September 1999 to 31 August 2000; post-MET implementation, 1 September 2000 to 31 October 2004. ^b^Plastics/Faciomaxillary/Ear nose and throat/Urology/Obstetrics and gynecology. PEA, pulseless electrical activity; SVT, supraventricular tachycardia; VF, ventricular fibrillation; VT, ventricular tachycardia.

**Table 3:** Predictors of death following cardiac arrest using multivariant logistic regression analysis

Variable	Odds ratio for death	95% confidence interval	*p *value
Male gender	2.88	1.34–6.19	0.007
Asystole	7.58	3.15–18.26	<0.0001
PEA	4.09	1.59–10.51	0.004
Cardiothoracic patient	0.15	0.05–0.49	0.002
Spinal injury patient	0.03	0.01–0.57	0.02
Cardiology patient	0.27	0.09–0.65	0.01
Arrest between 08:00 and 10:00 am	0.25	0.09–0.65	0.005

PEA, pulseless electrical activity.

## References

[B1] McGrath RB (1987). In-hospital cardiopulmonary resuscitation – after a quarter of a century. Ann Emerg Med.

[B2] Franklin C, Mathew J (1994). Developing strategies to prevent inhospital cardiac arrest: analyzing responses of physicians and nurses in the hours before the event. Crit Care Med.

[B3] Shein RM, Hazday N, Pena M, Ruben BH, Sprung CL (1990). Clinical antecedents to in-hospital cardiopulmonary arrest. Chest.

[B4] Kause J, Smith G, Prytherch D, Parr M, Flabouris A, Hillman K, Intensive Care Society (UK), Australian and New Zealand Intensive Care Society Clinical Trials Group (2004). A comparison of antecedents to cardiac arrests, deaths and emergency intensive care admissions in Australia and New Zealand, and the United Kingdom – the ACADEMIA study. Resuscitation.

[B5] Buist MD, Moore GE, Bernard SA, Waxman BP, Anderson JN, Nguyen TV (2002). Effects of a medical emergency team on reduction in incidence of and mortality from unexpected cardiac arrest in hospital: preliminary study. BMJ.

[B6] Kenward G, Castle N, Hodgetts T, Shaikh L (2004). Evaluation of a medical emergency team one year after implementation. Resuscitation.

[B7] Bellomo R, Goldsmith D, Uchino S, Buckmaster J, Hart GK, Opdam H, Silvester W, Doolan L, Gutteridge G (2003). A prospective before-and-after trial of a medical emergency team. Med J Aust.

[B8] DeVita MA, Braithwaite RS, Mahidhara R, Stuart S, Foraida M, Simmons RL, Medical Emergency Response Improvement Team (MERIT) (2004). Use of medical emergency team responses to reduce hospital cardioplumonary arrests. Qual Saf Health Care.

[B9] Firth-Cozens J (2003). Evaluating the culture of safety. Qual Saf Health Care.

[B10] Advances in Patient Safety: From Research to Implementation. http://www.ahrq.gov/qual/advances.

[B11] Foraida MI, DeVita MA, Braithwaite RS, Stuart SA, Brooks MM, Simmons RL (2003). Improving the utilization of medical crisis teams (condition C) at an urban tertiary care hospital. J Crit Care.

[B12] Bellomo R, Goldsmith D, Uchino S, Buckmaster J, Hart G, Opdam H, Silvester W, Doolan L, Gutteridge G (2004). Prospective controlled trial of effect of medical emergency team postoperative morbidity and mortality rates. Crit Care Med.

[B13] Hillman K, Chen J, Cretikos M, Bellomo R, Brown D, Doig G, Finfer S, Flabouris A, MERIT Study Investigators (2005). Introduction of the medical emergency team (MET) system: a cluster-randomised controlled trial. Lancet.

[B14] Jurkovich GJ (2000). Strengthening of the case for organized trauma-care systems. Lancet.

[B15] Bristow PJ, Hillman KM, Chey T, Daffum K, Jacques TC, Norman SL, Bishop GF, Simmons EG (2000). Rates of in-hosptial arrests, deaths and intensive care admissions: the effect of a medical emergency team. Med J Aust.

